# Free functional gracilis muscle transfer in children with severe sequelae from obstetric brachial plexus palsy

**DOI:** 10.1186/1749-7221-3-23

**Published:** 2008-10-30

**Authors:** Jörg Bahm, Claudia Ocampo-Pavez

**Affiliations:** 1 Euregio Reconstructive Microsurgery Unit, Franziskushospital, Aachen, Germany

## Abstract

We present 4 children between 6 and 13 years suffering from severe sequelae after a total obstetric brachial plexus lesion resulting in a hand without functional active long finger flexion. They had successfully reanimated long finger flexion using a free functional gracilis muscle transfer. These children initially presented a total obstetric brachial plexus palsy without neurotisation of the lower trunk in an early microsurgical nerve reconstruction procedure.

We describe our indications for this complex microsurgical procedure, the surgical technique and the outcome.

## Background

Obstetric brachial plexus palsy may result in a severe impairment of upper limb function. Early microsurgical reconstruction is proposed in upper and total palsies with insufficient functional recovery [1]. Nevertheless, major motor functions may not recover, both in operated or not operated children.

Free functional muscle transfer has been developed in the last 30 years to replace major muscle function, especially in the face and the upper limb [2,3]. Volkmann's ischemic contracture, tumor resection, and extensive palsy are possible indications.

An isolated motor deficit in a major upper limb function in children suffering from obstetric brachial plexus palsy might be corrected by means of a free muscle transfer, using the gracilis muscle. Finger and elbow flexion are obvious primary goals. These were also the indications where we decided to apply this technique.

We present our strategy, indications, operative technique and results.

We also report the advantages of this microsurgical procedure, but also technical drawbacks and reasonable limits of indication.

### Historical background [3]

The experimental background was set in 1970 when Tamai [4] reported the first successful transplantation of a rectus femoris muscle to the forelimb of a dog, using microneurovascular techniques.

The first clinical case was published 3 years later, when Chinese surgeons [5] transplanted part of a pectoralis major muscle to improve the hand function of a patient with Volkmann's ischemic contracture.

Harii [6] started to use the technique for a paralyzed face, Manktelow [7] applied it to the forearm region, Zuker [8] for children.

In the field of brachial plexus reconstruction, Doi [9] presented a new approach using two free gracilis muscle transfers to reconstruct major upper limb motors, and an extensive and impressive clinical series in children was recently published by Chuang [10].

### Patients

Our clinical series includes 900 children with obstetric brachial plexus palsy treated in our unit between 1997 to 2007, and about 150 microsurgical plexus reconstructions. In 7 cases suffering very severe total brachial plexus palsy without recovery of functional active long finger flexion (all 7), we performed a free gracilis muscle transfer, six for long finger flexion, one for biceps replacement.

A long-term follow-up is available in 4 children where the gracilis muscle replaced the long finger flexors, for whom we present the clinical background (table 1). The children had been initially treated elsewhere, and did not undergo early exploration and/or early microsurgical reconstruction, focusing specially on the lower trunk. They were operated in our Unit between 6 and 13 years.

**Table 1:** Clinical background of the operated children

I.	10 months	brachial plexus exploration, reconstruction of the upper and middle trunk, neurolysis of the lower trunk.
	3 years	extraplexic neurotisation SAN to ulnar nerve, without success
II.	3 months	Normal MRI, EMG shows only reinnervation of arm muscles, no recovery within forearm and hand.
	13 months	MyeloCT: suspicion of root avulsions C6 C8 Th1

III.		no previous surgery

IV.	14 months:	MyeloCT: suspicion of root avulsion C7 C8
		EMG: no activity C6 C7 C8 Th1
	28 months:	plexus exploration, only neurolysis C5

Before surgery, all children had recovered active wrist and finger extension, which are both mandatory requirements prior to surgery to stabilize the wrist and allow finger release. Otherwise, the transplanted muscle would rather flex the wrist and finger flexion would be weak.

**Figure 1:**
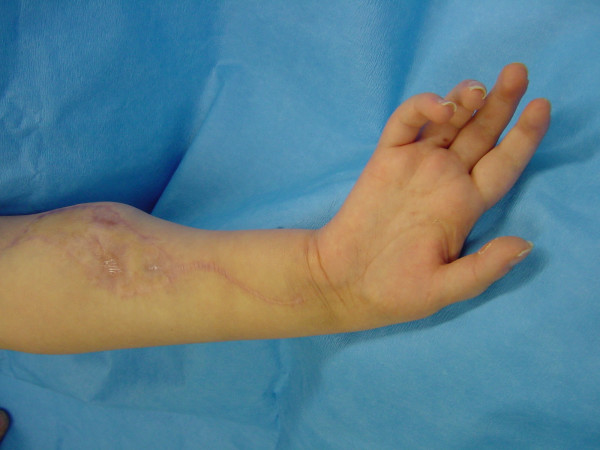
Preoperative flail hand.

Protective sensation was recovered, but the limb integration was poor due to poor motor function of the hand (figure 1; see additional file 1). All cases had a functional IP-joint flexion of the thumb, so we concentrated our flexor reconstruction on the long fingers.

Due to the extensive palsy, no local (forearm) muscles were available for a tendon or muscle transfer to improve the finger flexion.

### Examination methods, video recording

All children had a complete motor assessment before and after surgery. All surgeries were carried out once the anaesthesiologist had independently checked the children, especially for airway infections or fever of unknown origin (both criteria excluded a long lasting elective surgery under general anesthesia).

Video-recording was performed before and after surgery.

### Surgical technique

There were always three operative steps: The motor nerve donor (spinal accessorius nerve SAN) was identified and prolonged to the volar forearm level by a sural graft.

To make sure that nerve regeneration had occurred down to the distal end of the transplanted nerve, one or more nerve biopsies were taken to analyse the proportion and quality of newly myelinated nerve fibers. Only when the neuropathologist was convinced with a good regenerative capacity ie a substantial proportion of newly myelinated fibers, than the microsurgical transfer was performed in a 2 team approach.

### Step1: preparing the motor nerve

The SAN is identified through a routine supraclavicular transverse approach. The nerve is identified lateral to the brachial plexus area, in the subcutaneous space and followed down to its entrance into the trapezius muscle. The first motor collateral branch for the horizontal muscle part is spared, and the second larger branch running down is sectioned as distally as possible. An autologous sural nerve is harvested by routine step-cut incisions on the posterior leg and then coapted to the SAN by 10/0 microsutures in an antidromic direction after having passed the graft through a subcutaneous tunnel in the anterior arm.

The distal end of the sural nerve is buried in the muscle remnants at the proximal volar forearm level, and two non absorbable stitches mark the level of the nerve – muscle coaptation.

The arm is immobilized for 10 days in a sling and the child is discharged after 3 days.

### Step2: nerve biopsy

After 8 to 12 months, a nerve biopsy is performed at the proximal forearm level and sent for neuropathological examination. Slices are examined to determinate the proportion and quantity of myelinated fibers.

If the biopsy reveals an insufficient reinnervation quality, a new biopsy is conducted 3 months later, and meanwhile the nerve is buried again in the muscular bulk.

### Step3: free functional muscle transfer in 2 teams

**Figure 2:**
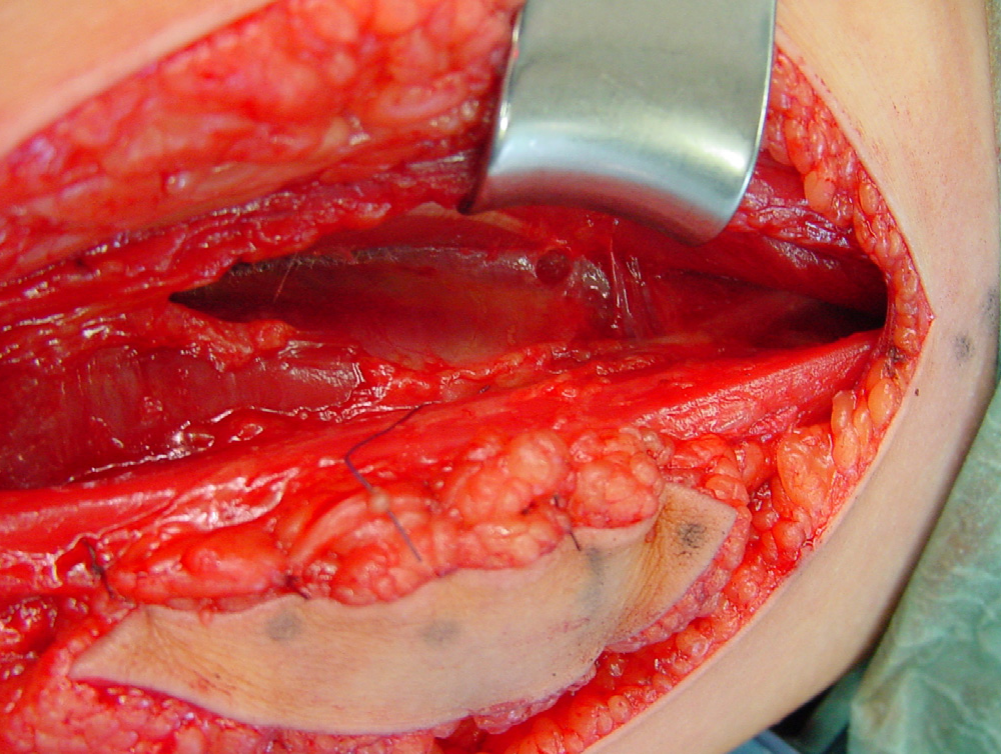
Intraoperative situation – gracilis muscle in the thigh.

**Figure 3:**
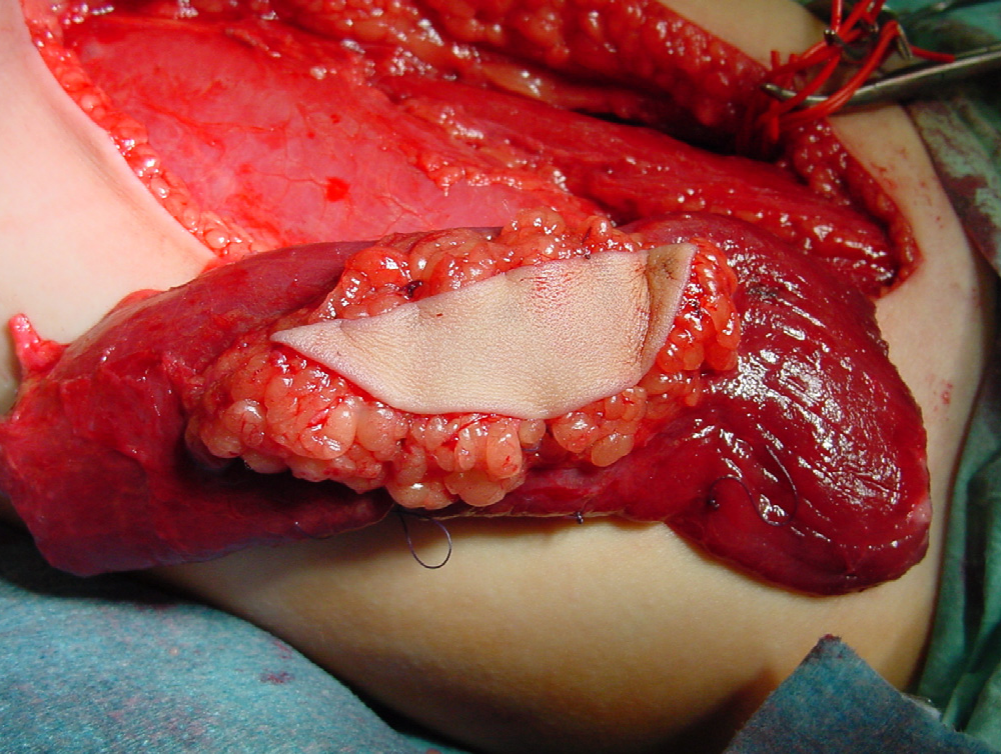
Intraoperative situation – muscle dissected free.

This day-lasting surgery is conducted in 2 teams; the first harvesting the gracilis muscle at the medial homolateral thigh (figures 2 and 3) according to Manktelow's technique [2], including a proximal and vertical skin monitor island and the anterior muscle aponeurosis to ensure better gliding; the second preparing the recipient site within the volar forearm, identifying the proximal vessels (collateral of the anterior interosseous artery, one or two superficial veins, the sural nerve graft end, and the deep finger flexors bundled together by side-to-side sutures in an harmonic finger adjustment, like in a natural fist (with slightly increasing finger flexion from the index to the little finger).

When the recipient site is ready with its vessels well prepared, the muscle harvest is completed by the division of the neurovascular bundle of the gracilis muscle and ischemia time is documented.

**Figure 4:**
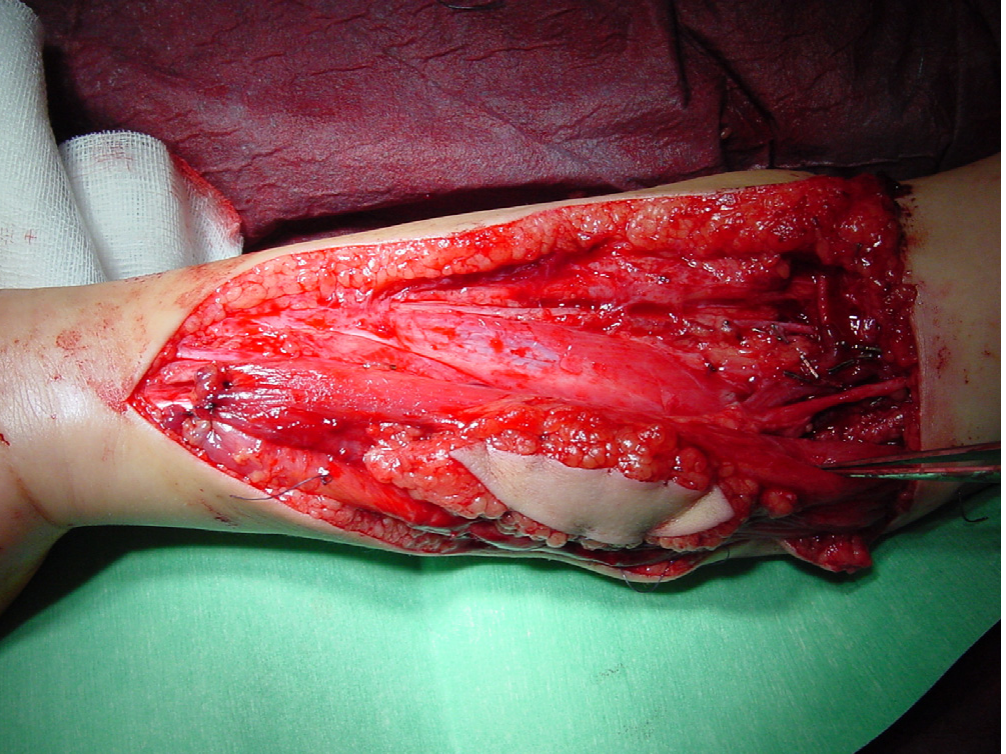
Intraoperative situation – muscle sutured in the forerarm.

**Figure 5:**
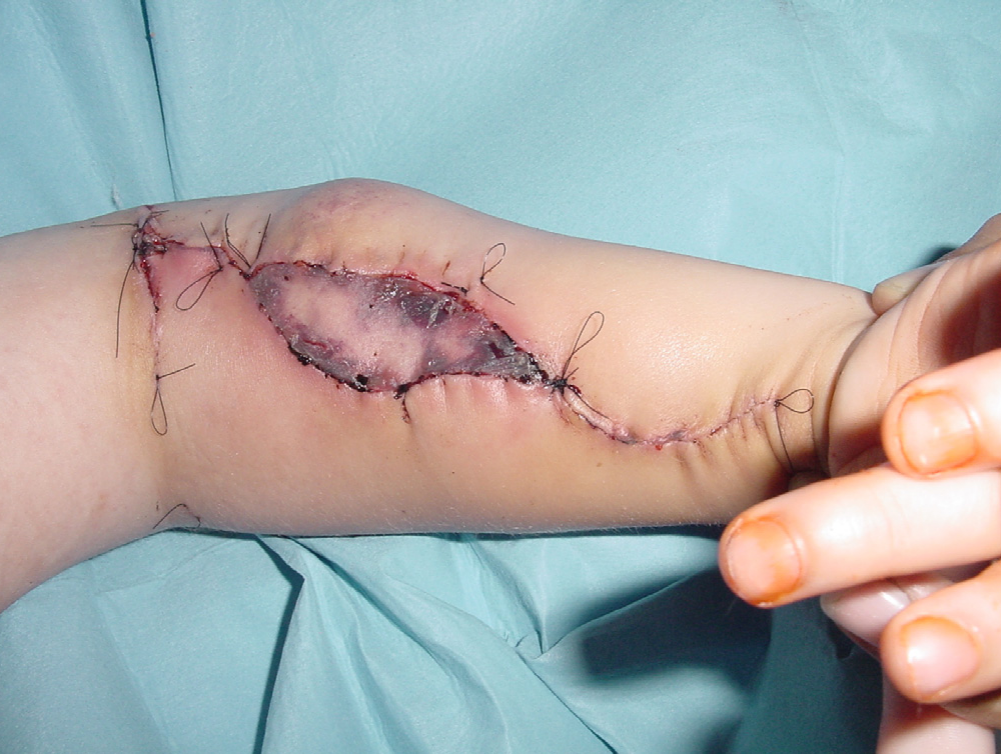
Intraoperative situation – closure.

Arterial and venous anastomosis are performed first, routinely as end to end coaptations, and then the nerve is sutured. Finally, the muscle tension is set using Manktelow's technique [2] of equidistant marker stitches and the transferred muscle is sutured within the medial epicondyle and the flexor tendons are anchored with a Pulvertaft-like technique within the distal muscle end which has been stabilized before by several absorbable stay-sutures (figure 4). Finally, wound closure is performed and the monitor skin island fixed with running intracutaneous sutures (figure 5).

The flexed fingers and the wrist in neutral position are immobilized in a dorsal forearm splint for 6 weeks.

We realized the free functional muscle transfer once the nerve biopsy showed sufficient regeneration, i.e. 9–14 months after the sural nerve transplant.

Every patient underwent 4 weeks postoperatively an angioMRI to check good vascular supply into the transposed muscle and an elective EMG at about 6 months.

The decision for the free muscle transfer was taken after one nerve biopsy in all children but one, where a second biopsy was mandatory six months later to show a sufficient reinnervation pattern. The first child had previously a SAN to ulnar nerve neurotisation at the proximal forearm level using a saphenous graft. In this case, the biopsy and the later nerve coaptation were made directly on the distal graft which was separated from the ulnar nerve.

In one other child, we observed one progressive monitor skin necrosis starting on the second day after surgery. Reexploration showed a patent vascular anastomosis and viable muscle tissue, so the skin island was removed.

In the other cases, no vascular disturbances were seen in the follow- up.

The angioMRI showed good vascularisation of the transferred muscle, before contractile activity begun.

Active finger flexion was noticed by the parents as soon as 6–8 months postoperatively and increased without specific training (table 2).

**Table 2:** Results after free gracilis muscle transfer

Delay to beginning muscle function:	6–8. months
Follow-up:	24. months
Function (global grasp):	M3 in 3 of 4 children

**Figure 6:**
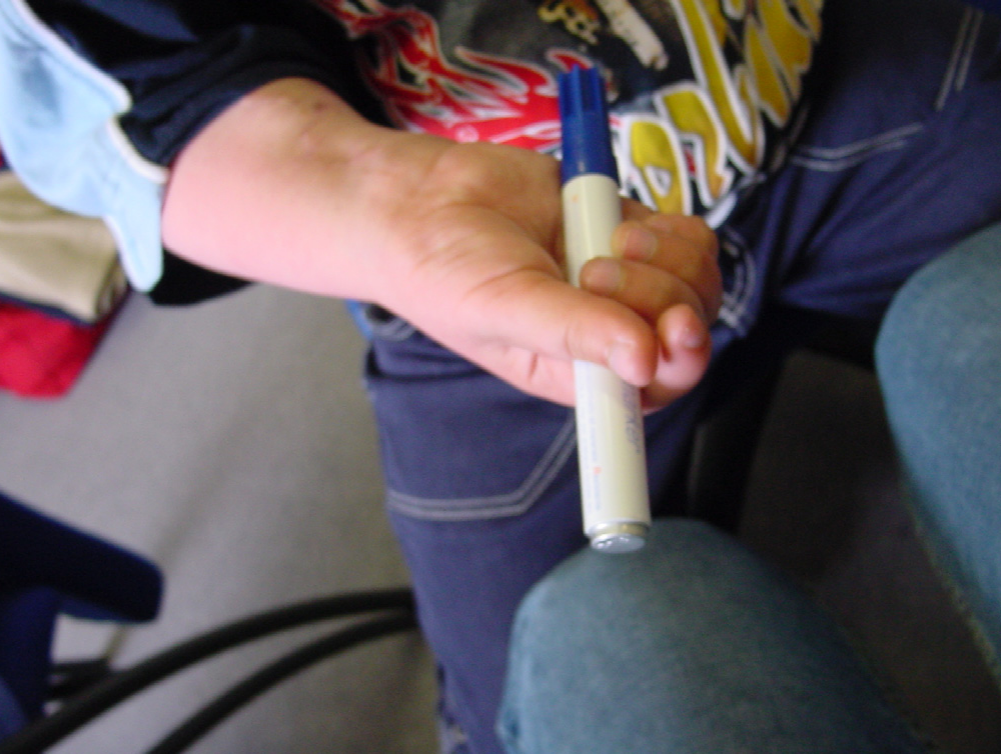
Postoperative function.

3 out of the 4 children obtained an equally good pattern of global finger flexion, as documented in figure 6.

Obviously, the reanimated hand remained a "helping hand", but a real grasp was now possible for the first time of their life (figure 6; see additional file 2).

For a mean follow-up of 2 years, we observed no weakness in the successfully reanimated hands.

There was one failure, and we reoperated that child 18 months later. The muscle was still present, but had undergone hypotrophy. We believe that either the regenerative capacity of the nerve graft or the nerve coaptation were insufficient. The remaining atrophied muscle tissue was well vascularised, although the pedicle could not be identified clearly. This child is actually scheduled for a new transfer.

## Discussion

Free functional muscle transfer is actually a rewarding procedure in selected indications, both in children and adults [3,9,10].

Other teams published successful cases, but details on results are rarely mentioned. Zuker and Manktelow [3] have experience in 30 forearm reconstructions with beginning muscle contraction as soon as 2 months after surgery. More than half of their patients were able to make a fist completely. The distal palmar crease-to-fingertip distance ranged from 0.5 to 4 cm in adults and was less good in children. Grip strength reached 38% of the normal side but 25% in children.

A good motor nerve connected with the shortest distance to a healthy and strong muscle which is adapted to the functional demand may result in a successfully reanimated major limb function. The reinnervation quality of the donor nerve could be followed by a progressing Hoffmann-Tinel sign along the anterior arm, but a qualitative assessment is only possible through histopathologic studies. The possibility of quantitative assessment of motor fiber content in the donor nerve is still under discussion [11].

Technical considerations in pediatric microsurgery refer to the vessel diameter (which might be 0.6 mm), the intraoperative conditions of fluid balance, blood tension and temperature (as in adult free flaps) and the technique of vascular and neural (micro)anastomosis, using 10/0 sutures.

As we observed one skin island necrosis with patent vascular anastomosis, obviating an unreliable monitor island, we furtheron include the whole fascia around the gracilis muscle as described by Addosooki et al [12] who reported on a 100% reliable skin monitor island.

Indications for this complex reconstructive procedure must be carefully established together with the child and the parents. Eventual alternatives, like pedicled muscle flaps, eg the brachialis muscle transfer described by Bertelli [13] must be discussed and risks and drawbacks must be outlined.

The donor site morbidity for the muscle harvest is minor, but the choice of the donor nerve might be critical, as other collegues would not hesitate to take the whole phrenic nerve to neurotise a free gracilis transfer even in young children and for a "minor" motor function like active finger extension [10]. Although this shows evidence of a high level of technical expertise, our general and cultural background would forbid these reconstructive procedures, where a reanimated minor function (eg wrist or finger extension) is reestablished harvesting a main motor nerve and damaging a major vital muscle like the hemidiaphragm in a young and growing child.

## Further developments

Neuropathologic assessment of the regenerative quality of the transplanted nerve by new techniques using a reliable motor fiber assessment (choline acetyl transferase activity) [11] could help to increase the prognostic value.

The knowledge of this microsurgical option should increase in the general pediatric and orthopaedic community, as it may be the only true solution to enhance function in a really useful manner.

The microsurgical risk remains a constant problem due to anatomical variations and technical failures.

## Competing interests

The authors declare that they have no competing interests.

## Authors' contributions

JB wrote the manuscript and was the responsible surgeon, COP corrected the manuscript and assisted in the surgeries. Both read and approved the final manuscript.

## Supplementary Material

Additional File 1 **Preoperative finger flexion.** This video shows the useless preoperative long finger flexion of the involved hand. Click here for file

Additional File 2 **Postoperative finger flexion.** This video shows the improvement in global long finger flexion making a global fist. Click here for file
